# Combined Therapy with Extracorporeal Shock Wave and Adipose-Derived Mesenchymal Stem Cells Remarkably Improved Acute Ischemia-Reperfusion Injury of Quadriceps Muscle

**DOI:** 10.1155/2018/6012636

**Published:** 2018-04-02

**Authors:** Tsung-Cheng Yin, Re-Wen Wu, Jiunn-Jye Sheu, Pei-Hsun Sung, Kuan-Hung Chen, John Y. Chiang, Shu-Kai Hsueh, Wen-Jung Chung, Pao-Yuan Lin, Shan-Ling Hsu, Chien-Chang Chen, Chen-Yu Chen, Pei-Lin Shao, Hon-Kan Yip

**Affiliations:** ^1^Department of Orthopaedic Surgery, Kaohsiung Chang Gung Memorial Hospital and Chang Gung University College of Medicine, Kaohsiung 83301, Taiwan; ^2^Division of Thoracic and Cardiovascular Surgery, Department of Surgery, Kaohsiung Chang Gung Memorial Hospital and Chang Gung University College of Medicine, Kaohsiung 83301, Taiwan; ^3^Division of Cardiology, Department of Internal Medicine, Kaohsiung Chang Gung Memorial Hospital and Chang Gung University College of Medicine, Kaohsiung 83301, Taiwan; ^4^Department of Anesthesiology, Kaohsiung Chang Gung Memorial Hospital and Chang Gung University College of Medicine, Kaohsiung 83301, Taiwan; ^5^Department of Computer Science and Engineering, National Sun Yat-sen University, Kaohsiung, Taiwan; ^6^Department of Plastic and Reconstructive Surgery, Kaohsiung Chang Gung Memorial Hospital and Chang Gung University College of Medicine, Kaohsiung 83301, Taiwan; ^7^Department of Hyperbaric Oxygen Therapy Center, Kaohsiung Chang Gung Memorial Hospital and Chang Gung University College of Medicine, Kaohsiung 83301, Taiwan; ^8^Department of Nursing, Asia University, Taichung 41354, Taiwan; ^9^Institute for Translational Research in Biomedicine, Kaohsiung Chang Gung Memorial Hospital, Kaohsiung 83301, Taiwan; ^10^Center for Shockwave Medicine and Tissue Engineering, Kaohsiung Chang Gung Memorial Hospital, Kaohsiung 83301, Taiwan; ^11^Department of Medical Research, China Medical University Hospital, China Medical University, Taichung 40402, Taiwan

## Abstract

Extracorporeal shock wave (ECSW) and adipose-derived mesenchymal stem cells (ADMSCs) have been recognized to have capacities of anti-inflammation and angiogenesis. We tested the hypothesis that ECSW and ADMSC therapy could attenuate ischemia-reperfusion- (IR-) induced thigh injury (femoral artery tightened for 6 h then the tightness was relieved) in rats. Adult male SD rats (*n* = 30) were divided into group 1 (sham-control), group 2 (IR), group 3 (IR + ECSW/120 impulses at 0.12 mJ/mm^2^ given at 3 h/24 h/72 h after IR), group 4 (allogenic ADMSC/1.2 × 10^6^ cell intramuscular and 1.2 × 10^6^ cell intravenous injections 3 h after IR procedure), and group 5 (ECSW + ADMSC). At day 7 after the IR procedure, the left quadriceps muscle was harvested for studies. At 18 h after the IR procedure, serum myoglobin/creatine phosphokinase (CPK) levels were highest in group 2, lowest in group 1, and with intermediate values significantly progressively reduced in groups 3 to 5 (all *p* < 0.0001). By day 5 after IR, the mechanical paw-withdrawal threshold displayed an opposite pattern of CPK (all *p* < 0.0001). The protein expressions of inflammatory, oxidative-stress, apoptotic, fibrotic, DNA-damaged, and mitochondrial-damaged biomarkers and cellular expressions of inflammatory and DNA-damaged biomarkers exhibited an identical pattern of CPK among the five groups (all *p* < 0.0001). The microscopic findings of endothelial-cell biomarkers and number of arterioles expressed an opposite pattern of CPK, and the angiogenesis marker was significantly progressively increased from groups 1 to 5, whereas the histopathology showed that muscle-damaged/fibrosis/collagen-deposition areas exhibited an identical pattern of CPK among the five groups (all *p* < 0.0001). In conclusion, ECSW-ADMSC therapy is superior to either one applied individually for protecting against IR-induced thigh injury.

## 1. Introduction

Rhabdomyolysis is a clinical syndrome characterized by muscle necrosis and loss of muscle function, resulting in the intracellular muscle constituents (e.g., electrolytes, myoglobin, creatine kinase, aldolase, lactate dehydrogenase, alanine aminotransferase, and aspartate aminotransferase) released into the circulation [1, 2]. The causal etiology of acute rhabdomyolysis is multifactorial, including those of acute ischemia-reperfusion (IR) injury, strenuous exercise, anesthesia, drug- or toxin-induced myopathies, muscle compression (e.g., crush syndrome or prolonged immobilization), hyperthermia, metabolic myopathies, electrolyte disorders, or even viral infections [1–8].

Increased circulating levels of myoglobin and creatine phosphokinase (CPK) (i.e., CK-MM form) are common biomarkers found after acute rhabdomyolysis. Additionally, the clinical manifestations and diagnosis of rhabdomyolysis include acute kidney injury and related metabolic complications. Furthermore, an increased intracellular calcium leads to the activation of proteases, increasing contractility of skeletal muscle cell, mitochondrial dysfunction and depletion of adenosine triphosphate (ATP) (i.e., results attributed to the dysfunction of the Na/K-ATPase and Ca2+-ATPase pump), and increase of reactive oxygen species (ROS) production as well as inflammatory reaction, resulting in skeletal muscle cell death [8].

Clinical observational study has shown that acute kidney injury (an estimated incidence from 13% to over 50% depending on both the cause and the clinical and organizational setting where they are diagnosed) [9] which is a serious complication of acute rhabdomyolysis will cause an unacceptably high morbidity and mortality [1] event undergoing the aggressive treatment such as decompression of compartment syndrome, restoration of ischemic etiology, diuretic agents, antioxidant therapy, or renal replacement therapy [10]. Accordingly, developing an alternative option with safety and efficacy is of paramount importance for physicians and patients.

Extracorporeal shock wave (ECSW) therapy is currently applied widely to muscle-skeletal disorders and rehabilitative medicine [11–14]. Additionally, studies have shown that ECSW plays a crucial role in regenerative medicine [15–18]. Moreover, growing data have demonstrated that ECSW has anti-inflammation angiogenesis properties [15, 17–20]. Interestingly, abundant data have shown that mesenchymal stem cells (MSCs), especially those of adipose-derived MSCs (ADMSCs), have strong capacity of anti-inflammation and immunomodulation as well as suppression of oxidative stress [21–23]. Based on the aforementioned observation, we tested the hypothesis that combined ECSW and ADMSC therapy would be superior to either one applied individually for protecting the thigh from IR injury in rodents.

## 2. Materials and Methods

### 2.1. Animals and Ethics Statement

All animal experimental protocols and procedures were approved by the Institute of Animal Care and Use Committee at Kaohsiung Chang Gung Memorial Hospital (Affidavit of Approval of Animal Use Protocol no. 2016032901) and performed in accordance with the Guide for the Care and Use of Laboratory Animals [The Eighth Edition of the Guide for the Care and Use of Laboratory Animals [24]].

Animals were housed in an Association for Assessment and Accreditation of Laboratory Animal Care International- (AAALAC-) approved animal facility in our hospital with controlled temperature and light cycles (24°C and 12/12 light cycle).

### 2.2. Animal Model of Limb Ischemia-Reperfusion (IR) Injury and Grouping

Pathogen-free, adult male Sprague–Dawley (SD) rats weighing 320–350 g (Charles River Technology, BioLASCO, Taiwan) were utilized in the present study. The procedure and protocol of limb IR injury in rodents were based on our previous report [25] with some modifications. In details, rats were anesthetized with inhalational 2.0% isoflurane and placed supine on a warming pad at 37°C with the left inguinal and thigh areas shaved. Under sterile conditions, small arterioles and the circumferential femoral artery were exposed over their proximal and distal portions and then were removed. To avoid the presence of collateral circulation, the branches were also removed. However, the veins were left intact during the procedure. On the other hand, the left femoral artery was tightened for 6 hours, then the tightness was relieved for reperfusion (i.e., to create an IR injury model for testing our hypothesis). For sham-control animals, only the skin and muscle layers of the left thigh were opened, followed by closure of these two layers. The animals in each group were euthanized by day 7 after the IR procedure, and the left quadriceps was harvested for individual study; that is, the ischemic time was 6 hours and the reperfusion time was 6 days and 18 hours.

After the procedure, a total amount of thirty animals (*n* = 30) were equally categorized into sham-control (SC) (*n* = 6), IR (*n* = 6) (treated by intramuscular injection of 0.3 cc culture medium), IR + ECSW (*n* = 6) (120 impulses at 0.12 mJ/mm^2^ given at 3 h, 24 h, and 72 h after IR), allogenic ADMSC (*n* = 6) (1.2 × 10^6^ cell intramuscular and 1.2 × 10^6^ cell intravenous injections 3 h after IR procedure), and combined ECSW + ADMSC (*n* = 6). The energy dosage of ECSW (120 impulses at 0.12 mJ/mm^2^) utilized in this study was based on our recent reports [17–19]. The number of ADMSC used in the present study was in accordance with other studies of ours [21–23].

### 2.3. Isolation of ADMSCs

For prepared allogenic ADMSCs, an additional 12 SD rats were utilized in the current study. The adipose tissue surrounding the epididymis was dissected, excised, and prepared based on our recent reports [21–23]. The adipose tissue was carefully cut into <1 mm^3^-size pieces using a pair of sharp, sterile surgical scissors followed by the addition of stock collagenase solution to a final concentration of 0.5 units/mL. The centrifuge tubes with the contents were placed and secured on a Thermaline shaker and incubated with constant agitation for 60 minutes at 37°C. After incubation for 40–45 minutes, the contents were triturated with a 25 mL pipette for 3 minutes. The isolated cells were put back into the rocker for incubation. The contents of the flask were transferred to 50 mL tubes after digestion, followed by centrifugation at 600*g* for 5 minutes at room temperature. The cell pellet thus obtained was resuspended in 40 mL saline and then centrifuged again at 600*g* for 5 minutes at room temperature. After being resuspended again in 5 mL saline, the cell suspension was filtered through a 100 *μ*m filter into a 50 mL conical tube to which 2 mL of saline was added to rinse the remaining cells through the filter. The flow-through was pipetted into a new 50 mL conical tube through a 40 *μ*m filter. The tubes were centrifuged for a third time at 600*g* for 5 minutes at room temperature. The cells were resuspended in saline. An aliquot of cell suspension was then removed for cell culture in Dulbecco's modified Eagle's medium- (DMEM-) low glucose medium containing 10% FBS for 14 days. Approximately 2.0–3.0 × 10^6^ ADMSCs were obtained from each rat.

### 2.4. Western Blot Analysis

Procedure was based on our recent reports [19, 21–23]. In details, equal amounts (50 *μ*g) of protein extracts were loaded and separated by SDS-PAGE using 8–12% acrylamide gradients. After electrophoresis, the separated proteins were transferred electrophoretically to a polyvinylidene difluoride (PVDF) membrane (Amersham Biosciences). Nonspecific sites were blocked by incubation of the membrane in blocking buffer [5% nonfat dry milk in T-TBS (TBS containing 0.05% Tween 20)] overnight. The membranes were incubated with the indicated primary antibodies [cleaved caspase 3 (1 : 1000, Cell Signaling), cleaved poly (ADP-ribose) polymerase (PARP) (1 : 1000, Cell Signaling), phosphorylated (p)-Smad3 (1 : 1000, Cell Signaling), p-Smad1/5 (1 : 1000, Cell Signaling), bone morphogenetic protein- (BMP-) 2 (1 : 500, Abcam), transforming growth factor- (TGF-) *β* (1 : 500, Abcam), cytosolic cytochrome C (1 : 1000, Millipore), mitochondrial cytochrome C (1 : 1000, Millipore), NOX-1 (1 : 1500, Sigma), NOX-2 (1 : 750, Sigma), *γ*-H2AX (1 : 1000, Cell Signaling), intercellular adhesion molecule- (ICAM-) 1 (1 : 1000, Abcam), matrix metalloproteinase- (MMP-) 9 (1 : 500, Abcam), tumor necrosis factor- (TNF-) *α* (1 : 1000, Cell Signaling), nuclear factor- (NF-) *κ*B (1 : 600, Abcam), regulated on activation, normal T cell expressed and secreted (RANTES) (1 : 1000, Cell Signaling), toll-like receptor- (TLR-) 2 (1 : 1000, Abcam), toll-like receptor- (TLR-) 4 (1 : 500, Abcam), and interleukin- (IL-) 1*β* (1 : 1000, Cell Signaling)] for 1 hour at room temperature. Horseradish peroxidase-conjugated anti-rabbit immunoglobulin IgG (1 : 2000, Cell Signaling) was used as the secondary antibody for one-hour incubation at room temperature. The washing procedure was repeated eight times within an hour, and immunoreactive bands were visualized by enhanced chemiluminescence (ECL; Amersham Biosciences) after exposure to Biomax L film (Kodak). For purposes of quantification, ECL signals were digitized using Labwork software (UVP).

### 2.5. Immunofluorescent (IF) Staining

The procedure and protocol of IF staining have been reported in details in our previous and recent reports [19, 21–23]. For IF staining, rehydrated paraffin sections were first treated with 3% H_2_O_2_ for 30 minutes and incubated with Immuno-Block reagent (Bio SB, Santa Barbara, CA, USA) for 30 minutes at room temperature. Sections were then incubated with primary antibodies specifically against CD31 (1 : 100, BIO-RAD, CA, USA), von Willebrand factor (vWF) (1 : 200, Millipore, Massachusetts, USA), Cox-2 (1 : 100, Abcam), CD68 (1 : 100, Abcam), and vascular endothelial growth factor (VEGF) (1 : 400, Abcam). Three sections of quadriceps specimen from each rat were analyzed. For quantification, three randomly selected high-power fields (HPFs) (400x for IF study) were analyzed in each section. The mean number of positively stained cells per HPF for each animal was then determined by summation of all numbers divided by 9.

### 2.6. Histological Study of Fibrosis and Condensed Collagen-Deposition Area

The procedure and protocol have been described in our previous reports [17, 26, 27]. In details, Masson's trichrome and Sirius red stainings were used for studying fibrosis and collagen deposition in quadriceps. Three 4 *μ*m-thick serial sections of quadriceps were prepared by Cryostat (Leica CM3050S). The integrated area (*μ*m^2^) of fibrosis in each section was calculated using ImageTool 3 (IT3) image analysis software (University of Texas, Health Science Center, San Antonio (UTHSCSA); ImageTool for Windows, Version 3.0, USA). Three selected sections were quantified for each animal. Three randomly selected HPFs (100x) were analyzed in each section. After determining the number of pixels in each fibrotic area per HPF, the numbers of pixels obtained from the three HPFs were summed. The procedure was repeated in two other sections for each animal. The mean pixel number per HPF for each animal was then determined by summing all pixel numbers and dividing by 9. The mean integrated area (*μ*m^2^) of fibrosis in quadriceps per HPF was obtained using a conversion factor of 19.24 (1 *μ*m^2^ corresponded to 19.24 pixels).

### 2.7. Vessel Density in Ischemic Quadriceps Muscle

Immunohistochemical staining of blood vessels was performed with alpha-smooth actin (*α*-SMA) (1 : 400) as the primary antibody at room temperature for one hour, followed by washing with phosphate buffer solution (PBS) thrice. The anti-mouse-HRP-conjugated secondary antibody was then added for 10 minutes, followed by washing with PBS thrice. Then 3,3′-diaminobenzidine (DAB) (0.7 gm/tablet) (Sigma) was added for one minute, followed by washing with PBS thrice. Finally, hematoxylin was added for one minute as a counter-stain for nuclei, followed by washing twice. For quantification, three sections of the ischemic area were chosen for each animal and three randomly selected HPF (100x) were analyzed for each section. The mean number per HPF for each animal was then determined by summation of all numbers divided by 9.

### 2.8. Statistical Analysis

Quantitative data are expressed as means ± SD. Statistical analysis was performed by ANOVA followed by Bonferroni multiple-comparison post hoc test. Statistical analysis was performed using SAS statistical software for Windows version 9.4 (SAS institute, Cary, NC). A probability value < 0.05 was considered statistically significant.

## 3. Results

### 3.1. Serum Levels of Myoglobin and CPK at 12 h and Mechanical Paw Withdrawal Threshold (MPWT) at Day 7 after IR Procedure (Figure 1)

At 12 h after the IR procedure, serum levels of myoglobin and CPK, two indicators of muscle damage/necrosis, were highest in the IR, lowest in the SC, and with intermediate values significantly progressively decreased from the IR-ECSW to the IR-ADMSC and to the IR + ECSW-ADMSC groups. Additionally, at day 5 after the IR procedure, MPWT was highest in the SC, lowest in the IR, significantly lower in the IR-ECSW than in the IR-ADMSC and the IR + ECSW-ADMSC, and significantly lower in the IR-ADMSC than the IR + ECSW-ADMSC groups, suggesting that our IR-induced quadriceps injury model was successfully created and the combined ECSW-ADMSC therapy more effectively protected the muscle from IR damage.

### 3.2. H&E Stain for Identification of Muscle Injury Area at Day 7 after IR Procedure (Figure 2)

The microscopic finding of H&E stain demonstrated that the quadriceps injury area was highest in the IR, lowest in the SC, significantly higher in the CLI + ECSW than in the CLI + ADMSC and CLI + ECSW-ADMSC, and significantly higher in the CLI + ADMSC than in the CLI + ECSW-ADMSC groups.

### 3.3. Histopathological Findings of Fibrotic and Condensed Collagen-Deposition Areas in the Quadriceps by Day 7 after IR Procedure (Figures 3 and 4)

**Figure 3:**
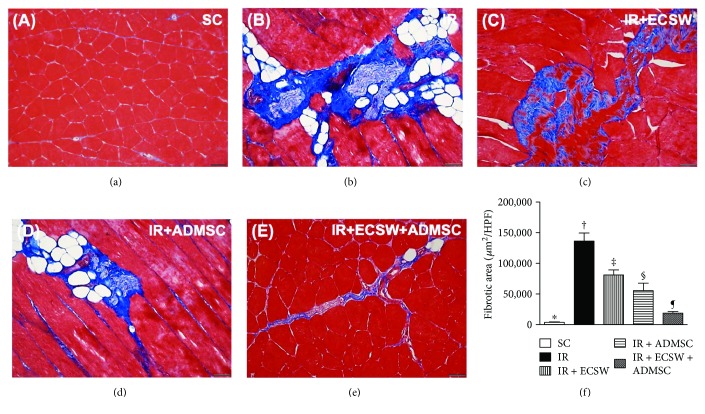
Histopathological finding of fibrotic area in the quadriceps by day 7 after the IR procedure. (a–e) Illustrating the microscopic finding (200x) of Masson's trichrome stain for identification of the fibrotic area of the quadriceps (blue color). (f) Analytic result of fibrotic area. ∗ versus other groups with different symbols (†, ‡, §, and ¶), *p* < 0.0001. The scale bars in the lower right corner represent 50 *μ*m. All statistical analyses were performed by one-way ANOVA, followed by Bonferroni multiple comparison post hoc test (*n* = 6 for each group). Symbols (∗, †, ‡, §, and ¶) indicate significance at the 0.05 level. HPF = high-power field; SC = sham control; IR = ischemia-reperfusion; ECSW = extracorporeal shock wave; ADMSC = adipose-derived mesenchymal stem cell.

**Figure 4:**
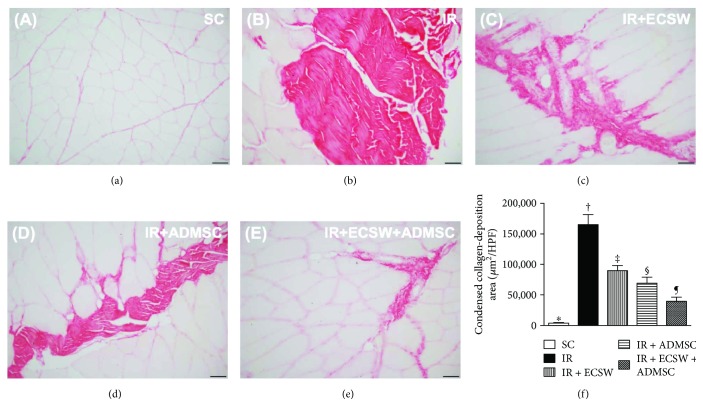
Histopathological finding of condensed collagen-deposition area in the quadriceps by day 7 after the IR procedure. (a–e) Illustrating the microscopic finding (200x) of Sirius red stain for identification of the condensed collagen-deposition area of the quadriceps (blue color). (f) Analytic result of condensed collagen-deposition area. ∗ versus other groups with different symbols (†, ‡, §, and ¶), *p* < 0.0001. The scale bars in the lower right corner represent 50 *μ*m. All statistical analyses were performed by one-way ANOVA, followed by Bonferroni multiple comparison post hoc test (*n* = 6 for each group). Symbols (∗, †, ‡, §, and ¶) indicate significance at the 0.05 level. HPF = high-power field; SC = sham control; IR = ischemia-reperfusion; ECSW = extracorporeal shock wave; ADMSC = adipose-derived mesenchymal stem cell.

The microscopic finding of Masson's trichrome stain exhibited that the fibrotic area of the quadriceps was highest in the IR, lowest in the SC, and with significantly progressively decreasing value from the IR-ECSW to the IR-ADMSC and then to the IR + ECSW-ADMSC groups (Figure 3). Additionally, the microscopic finding of Sirius red stain demonstrated that the condensed collagen-deposition area expressed an identical pattern of fibrosis among the five groups (Figure 4).

### 3.4. Angiogenesis Biomarkers and Small Vessel Density in the Quadriceps by Day 7 after IR Procedure (Figures 5 and 6)

**Figure 5:**
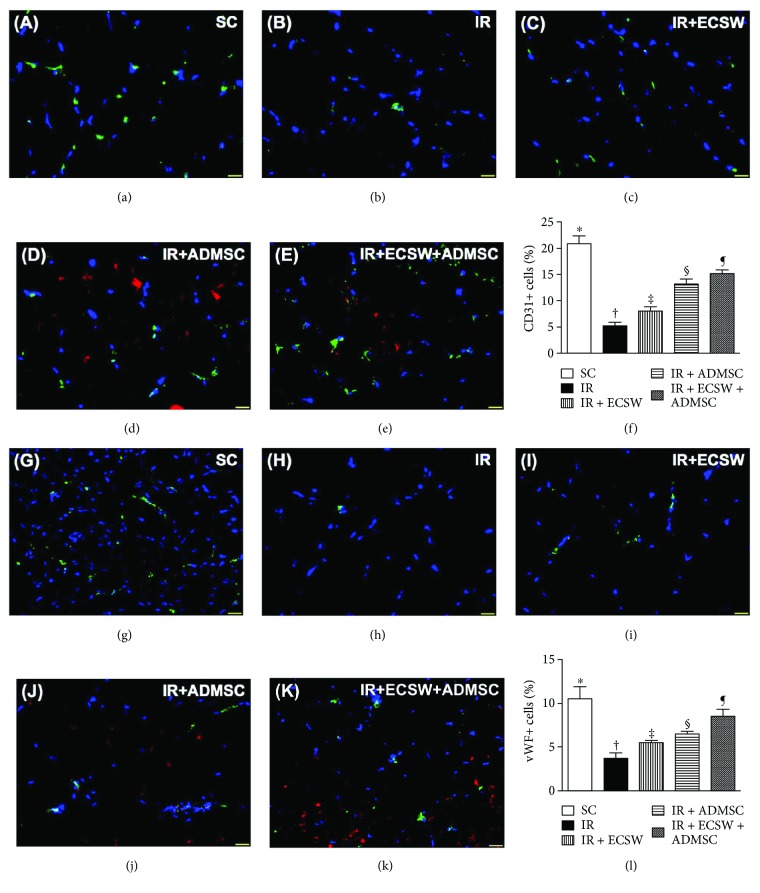
Angiogenesis biomarkers in the quadriceps by day 7 after the IR procedure. (a–e) Showing the immunofluorescent (IF) microscopic finding (400x) for identification of CD31+ cells in IR-injured quadriceps (green color). Red color indicated Dil-dye-stained ADMSCs. (f) Analytical result of number of CD31+ cells. ∗ versus other groups with different symbols (†, ‡, §, and ¶), *p* < 0.0001. (g–k) Showing the IF microscopic finding (400x) for identification of von Willebrand factor (vWF)+ cells in IR-injured quadriceps (green color). Red color indicated Dil-dye-stained ADMSCs. (l) Analytical result of number of vWF+ cells. ∗ versus other groups with different symbols (†, ‡, §, and ¶), *p* < 0.0001. The scale bars in the lower right corner represent 20 *μ*m. All statistical analyses were performed by one-way ANOVA, followed by Bonferroni multiple comparison post hoc test (*n* = 6 for each group). Symbols (∗, †, ‡, §, and ¶) indicate significance at the 0.05 level. SC = sham control; IR = ischemia-reperfusion; ECSW = extracorporeal shock wave; ADMSC = adipose-derived mesenchymal stem cell.

**Figure 6:**
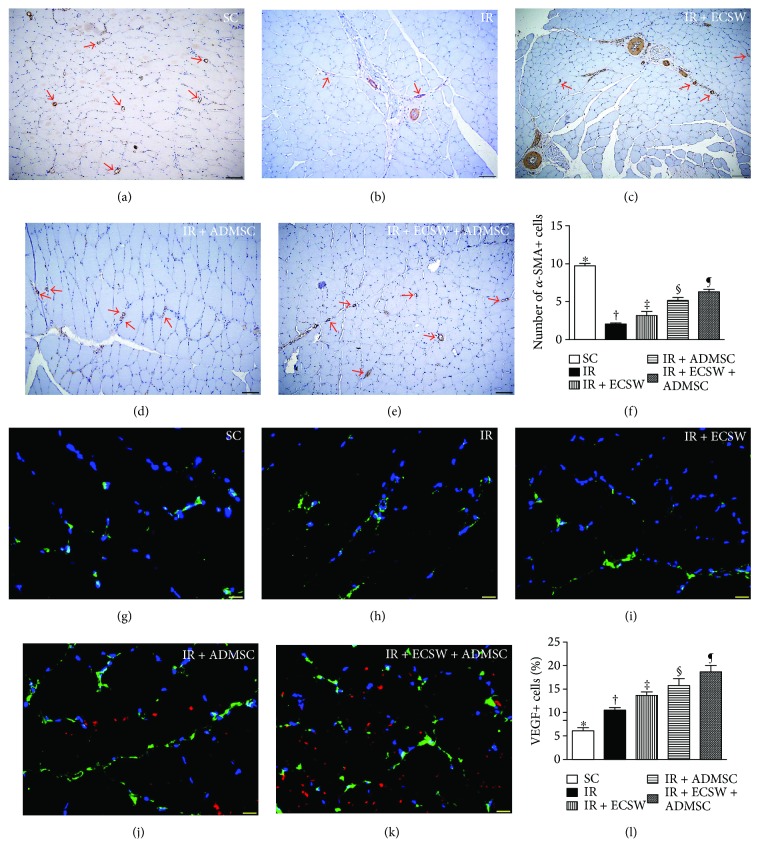
Small vessel density in the quadriceps by day 7 after the IR procedure. (a–e) Illustrating the microscopic finding (100x) of *α*-smooth muscle actin (SMA) for identification of small vessels (i.e., diameter ≤ 25 *μ*M) in IR-injured quadriceps (red arrows). (f) Analytical result of number of small vessels. ∗ versus other groups with different symbols (†, ‡, §, and ¶), *p* < 0.0001. The scale bars in the lower right corner represent 100 *μ*m. (g–k) Illustrating immunofluorescent (IF) microscopic finding (400x) for identification of vascular endothelial growth factor (VEGF)+ cells in IR-injured quadriceps (green color). Red color indicated Dil-dye-stained ADMSCs. (l) Analytical result of number of VEGF+ cells. ∗ versus other groups with different symbols (†, ‡, §, and ¶), *p* < 0.0001. The scale bars in the lower right corner represent 20 *μ*m. All statistical analyses were performed by one-way ANOVA, followed by Bonferroni multiple comparison post hoc test (*n* = 6 for each group). Symbols (∗, †, ‡, §, and ¶) indicate significance at the 0.05 level. SC = sham control; IR = ischemia-reperfusion; ECSW = extracorporeal shock wave; ADMSC = adipose-derived mesenchymal stem cell.

IF microscopic findings demonstrated that the numbers of CD31+ and vWF+ cells, two indicators of endothelial cell markers, were highest in the SC, lowest in the IR, and significantly progressively increased from the IR-ECSW to the IR-ADMSC and to the IR + ECSW-ADMSC groups (Figure 5). Additionally, the number of small vessels (i.e., diameter ≤ 25 *μ*M), an indicator of angiogenesis/neovascularization, displayed an identical pattern of CD31+ cells among the five groups. On the other hand, the IF microscopic finding revealed that the number of VEGF+ cells, another indicator of angiogenesis, was significantly progressively increased from the SC to the IR + ECSW-ADMSC group (Figure 6), suggesting an intrinsic response to ischemia and notably increased by ECSW-ADMSC treatment.

### 3.5. Cellular Expression of Inflammatory Biomarkers in the Quadriceps by Day 7 after IR Procedure (Figure 7)

The IF microscopic finding demonstrated that the numbers of Cox-2+ and CD68+ cells, two indicators of inflammation, were highest in the IR, lowest in the SC, while significantly progressively decreased from the IR-ECSW to the IR-ADMSC and to the IR + ECSW-ADMSC groups.

### 3.6. DNA and Mitochondrial Damaged Biomarkers in the Quadriceps by Day 7 after IR Procedure (Figure 8)

The protein expression of cytosolic cytochrome C, an indicator of mitochondrial damage, was highest in the IR, lowest in the SC, and significantly progressively decreased from the IR-ECSW to the IR-ADMSC and to the IR + ECSW-ADMSC groups. On the other hand, the protein expression of mitochondrial cytochrome C, an indicator of mitochondrial integrity, displayed an opposite pattern among the five groups.

The protein and cellular expressions of *γ*-H2AX, an indicator of DNA damage, followed an identical pattern of cytosolic cytochrome C among the five groups.

### 3.7. Protein Expressions of Inflammatory Biomarkers in the Quadriceps by Day 7 after IR Procedure (Figure 9)

The protein expression of ICAM-1, MMP-9, TNF-*α*, NF-*κ*B, RANTES, TLR-2, TLR-4, and IL-1*β*, eight indicators of inflammation, was highest in the IR, lowest in the SC, and significantly progressively decreased from the IR-ECSW to the IR-ADMSC and to the IR + ECSW-ADMSC groups.

### 3.8. Protein Expressions of Oxidative-Stress, Apoptotic, Antiapoptotic, Fibrotic, and Antifibrotic Biomarkers in the Quadriceps by Day 7 after IR Procedure (Figure 10)

The protein expressions of NOX-1 and NOX-2, two indicators of oxidative stress, were highest in the IR, lowest in the SC, and significantly progressively decreased from the IR-ECSW to the IR-ADMSC and then to the IR + ECSW-ADMSC groups. Additionally, protein expressions of cleaved caspase 3 and cleaved PARP, two indicators of apoptosis, and TGF-*β* and phosphorylated- (p-) Smad3, two indices of fibrosis, exhibited an identical pattern of oxidative stress among the five groups. On the other hand, the protein expressions of Bcl-2, an indicator of antiapoptosis, and p-Smad1/5 and BMP-2, two indices of antifibrosis, demonstrated an opposite pattern of oxidative stress among the five groups.

## 4. Discussion

This study which investigated the therapeutic impact of ECSW-ADMSC on attenuating the thigh from IR injury yielded several striking implications. Frist, serum levels of myoglobin and CPK were markedly increased in IR animals and notably decreased after receiving ECSW-ADMSC therapy, suggesting not only the animal model of acute quadriceps injury was successfully created but also the ECSW-ADMSC therapy was effective for protecting the muscle against IR injury. Second, not only the physical examinations (i.e., MPWT) but also the histopathological findings proved the successful creation of muscle injury by IR procedure and markedly reduced muscle damage by ECSW-ADMSC therapy, highlighting that ECSW-ADMSC therapy may be an alternative option for those patients with acute rhabdomyolysis refractory to conventional therapy. Third, inflammation, oxidative stress, fibrosis, and apoptosis/DNA damage were found to be closely associated with quadriceps damage that was substantially reduced in animals after receiving ECSW-ADMSC treatment.

Clinical observational studies have previously well documented that the peak level of CPK was identified at 24–72 h after acute muscle injury [28–30]. Additionally, a strong association between the circulating level and the severity of muscle injury has been established by these studies [28–30]. Furthermore, the circulating level of myoglobin is a useful biomarker for prediction of acute renal failure after muscle injury/rhabdomyolysis [31, 32] which not only elicits an overwhelming release of proinflammatory mediators/cytokines [33, 34] and the generation of oxidative stress but would also cause unacceptable high inhospital mortality [1]. One important finding in the present study was that as compared with SC, the serum levels of myoglobin and CPK were remarkably increased in the IR animals. However, these biomarkers were found to be significantly reduced in IR animals after receiving the ECSW or the ADMSC treatment and further notably reduced in IR animals treated by the combined ECSW-ADMSC regimen. Our findings, in addition to being comparable with those of clinical studies [28–32], highlight that ECSW-ADMSC therapy may have therapeutic potential for those patients with rhabdomyolysis complicated by acute renal failure who are refractory to traditional therapy.

Our previous study has demonstrated that ECSW therapy effectively protected the sciatic nerve against diabetic neuropathy [15]. Intriguingly, our recent study has shown that ECSW therapy markedly ameliorated neuropathic pain induced by chronic constriction injury [35]. An essential finding in the present study was that the ECSW or the ADMSC therapy individually significantly improved and the combined ECSW-ADMSC therapy even more significantly improved MPWT by day 5 after the IR procedure. The results of these physical examinations were supported by our previous studies [15, 35].

Interestingly, our previous study has shown either the ECSW or the ADMSC therapy significantly reduced fibrosis and collagen deposition in the ischemic area [17, 19, 21–23, 35]. Additionally, our other recent report has also found that combined ECSW and bone marrow-derived stem cells was superior to either one applied individually in improving ischemia in rodent critical limb ischemia [25]. The most important discovery in the present study was that the histopathological findings of muscle injury (i.e., pathological finding by H&E stain), fibrotic (i.e., by Masson's trichrome stain), and condensed collagen-deposition (i.e., by Sirius oil red stain) areas of the quadriceps were substantially higher in the IR animals than in the SC animals. Noteworthily, these histopathological parameters in the IR animals were markedly ameliorated by the ECSW or the ADMSC treatment. Of particular importance was that the combined ECSW and ADMSC therapy further prominently mitigated these histopathological parameters in the IR animals. Our findings, in addition to corroborating the findings of our previous studies [17, 19, 21–23, 25, 35], raise that ECSW-ADMSC may be an alternative modality for reducing the incidence of rhabdomyolysis in patients with setting of muscle ischemia/IR injury.

An association between IR injury and inflammatory reaction, generation of oxidative stress, mitochondrial/DNA damage, and cellular apoptosis has been keenly investigated by abundant previous studies [21, 23, 26, 36]. Additionally, the link between an increase in these aforementioned molecular-cellular perturbations and the severity of IR-induced organ injury has been extensively investigated in these previous reports [21, 23, 26, 36]. Interestingly, when we peruse the cellular-molecular examinations, we found that as compared with SC animals, the aforementioned molecular-cellular perturbations were remarkably increased in IR animals, while significantly reduced in IR animals after receiving the ECSW or the ADMSC treatment individually and further remarkably reduced in IR animals after receiving the combined ECSW-ADMSC treatment. Our findings, consistent with those of previous studies [21, 23, 26, 36], provide an insight regarding the MPWT, and the fibrosis and the collagen-deposition area were significantly preserved in the IR animals treated by ECSW-ADMSC.

### 4.1. Study Limitations

This study has limitations. First, the study period was relatively short (i.e., only 7 days). Accordingly, the long-term effect of ECSW-ADMSC for the regeneration of the quadriceps has not been investigated. Second, the parameters of acute kidney injury biomarkers, such as creatinine or blood urine nitrogen level, were not measured in the present study. Third, the assessment of optimal dosage of ECSW or ADMSC was not performed, so we did not know whether ADMSC is superior to ECSW or vice versa.

## 5. Conclusions

In conclusion, the results of the present study identified that combined ECSW and ADMSC therapy is superior to either one alone for alleviating the quadriceps from acute IR injury.

## Figures and Tables

**Figure 1:**
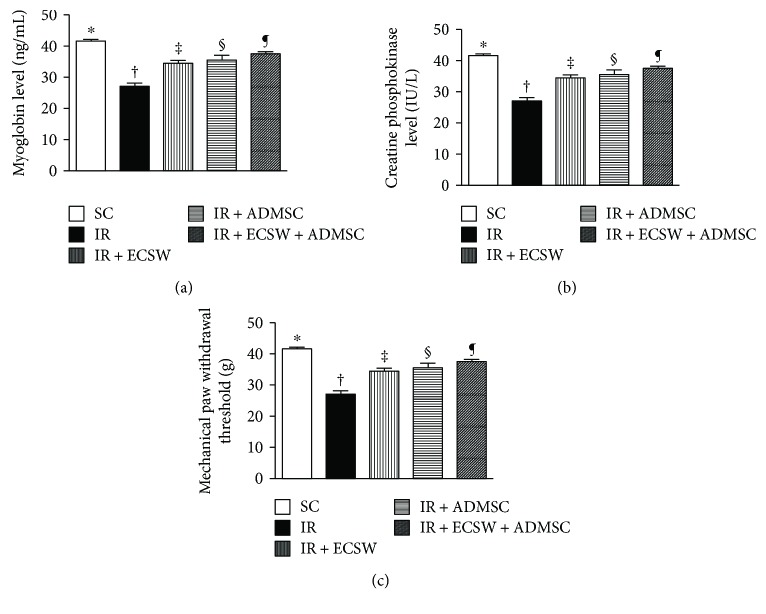
Circulating myoglobin and CPK at 12 h and mechanical paw withdrawal threshold (MPWT) at day 7 after the IR procedure. (a) Circulating level of myoglobin. ∗ versus other groups with different symbols (†, ‡, §, and ¶), *p* < 0.0001. (b) Circulating level of creatine phosphokinase (CPK). ∗ versus other groups with different symbols (†, ‡, §, and ¶), *p* < 0.0001. (c) The value of mechanical paw withdrawal threshold. ∗ versus other groups with different symbols (†, ‡, §, and ¶), *p* < 0.0001. All statistical analyses were performed by one-way ANOVA, followed by Bonferroni multiple comparison post hoc test (*n* = 6 for each group). Symbols (∗, †, ‡, §, and ¶) indicate significance at the 0.05 level. SC = sham control; IR = ischemia-reperfusion; ECSW = extracorporeal shock wave; ADMSC = adipose-derived mesenchymal stem cell.

**Figure 2:**
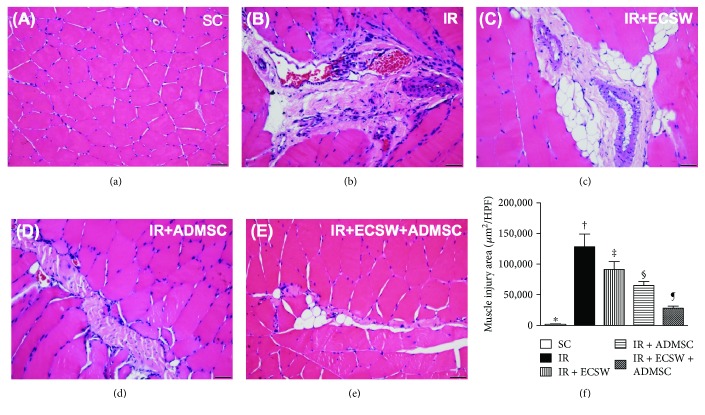
Muscle injury area at day 7 after the IR procedure (Figure 2). (a–e) Illustrating the microscopic finding (200x) of H&E stain for identification of quadriceps injured area. (f) Analytical result of the injured area. ∗ versus other groups with different symbols (†, ‡, §, and ¶), *p* < 0.0001. The scale bars in the lower right corner represent 50 *μ*m. All statistical analyses were performed by one-way ANOVA, followed by Bonferroni multiple comparison post hoc test (*n* = 6 for each group). Symbols (∗, †, ‡, §, and ¶) indicate significance at the 0.05 level. HPF = high-power field; SC = sham control; IR = ischemia-reperfusion; ECSW = extracorporeal shock wave; ADMSC = adipose-derived mesenchymal stem cell.

**Figure 7:**
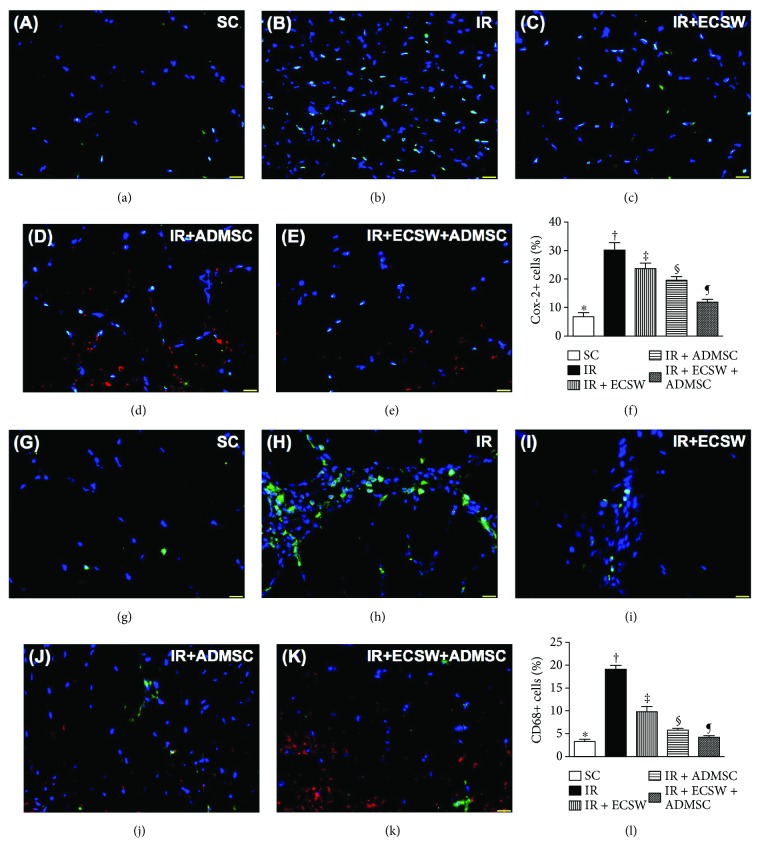
Cellular expression of inflammatory biomarkers in the quadriceps by day 7 after the IR procedure. (a–e) Showing the immunofluorescent (IF) microscopic finding (400x) for identification of Cox-2+ cells in IR-injured quadriceps (green color). Red color indicated Dil-dye-stained ADMSCs. (f) Analytical result of number of Cox-2+ cells. ∗ versus other groups with different symbols (†, ‡, §, and ¶), *p* < 0.0001. (g–k) Showing the IF microscopic finding (400x) for identification of CD68+ cells in IR-injured quadriceps (green color). Red color indicated Dil-dye-stained ADMSCs. (l) Analytical result of number of CD68+ cells. ∗ versus other groups with different symbols (†, ‡, §, and ¶), *p* < 0.0001. Scale bars in the lower right corner represent 20 *μ*m. All statistical analyses were performed by one-way ANOVA, followed by Bonferroni multiple comparison post hoc test (*n* = 6 for each group). Symbols (∗, †, ‡, §, and ¶) indicate significance at the 0.05 level. SC = sham control; IR = ischemia-reperfusion; ECSW = extracorporeal shock wave; ADMSC = adipose-derived mesenchymal stem cell.

**Figure 8:**
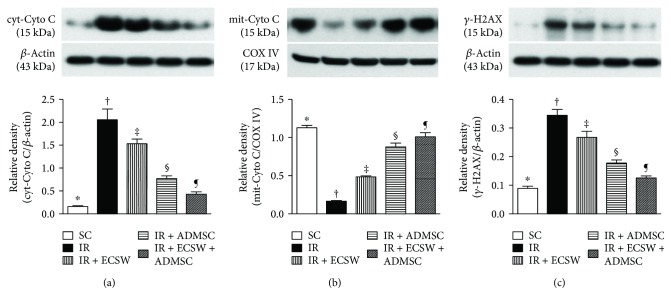
DNA and mitochondrial-damaged biomarkers in the quadriceps by day 7 after IR procedure. (a) Protein expression of cytosolic cytochrome C (cyt-Cyto C). ∗ versus other groups with different symbols (†, ‡, §, and ¶), *p* < 0.0001. (b) Protein expression of mitochondrial cytochrome C (mit-Cyto C). ∗ versus other groups with different symbols (†, ‡, §, and ¶), *p* < 0.0001. (c) Protein expression of *γ*-H2AX. ∗ versus other groups with different symbols (†, ‡, §, and ¶), *p* < 0.0001. All statistical analyses were performed by one-way ANOVA, followed by Bonferroni multiple comparison post hoc test (*n* = 6 for each group). Symbols (∗, †, ‡, §, and ¶) indicate significance at the 0.05 level. SC = sham control; IR = ischemia-reperfusion; ECSW = extracorporeal shock wave; ADMSC = adipose-derived mesenchymal stem cell.

**Figure 9:**
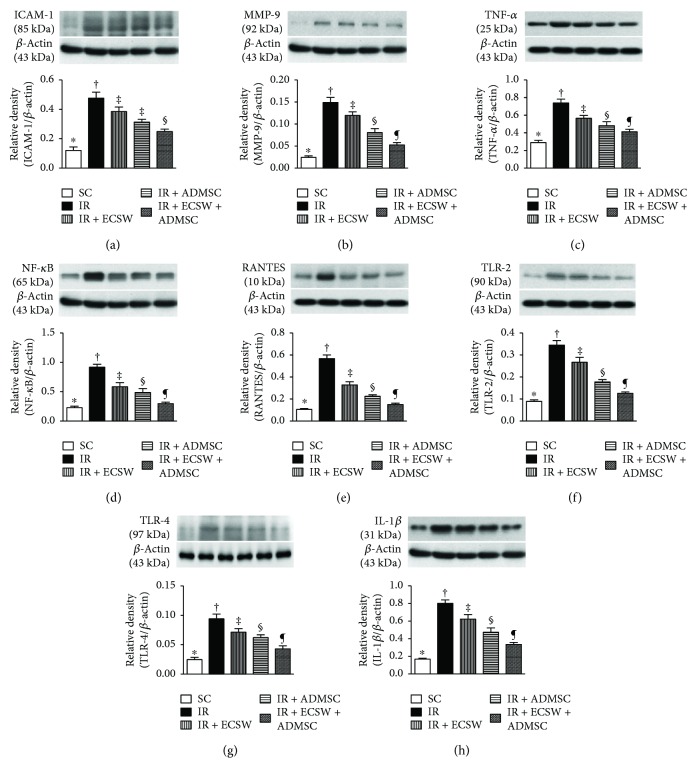
Protein expressions of inflammatory biomarkers in the quadriceps by day 7 after the IR procedure. (a) Protein expression of intercellular adhesion molecule- (ICAM-) 1. ∗ versus other groups with different symbols (†, ‡, §, and ¶), *p* < 0.0001. (b) Protein expression of matrix metalloproteinase- (MMP-) 9. ∗ versus other groups with different symbols (†, ‡, §, and ¶), *p* < 0.0001. (c) Protein expression of tumor necrosis factor- (TNF-) *α*. ∗ versus other groups with different symbols (†, ‡, §, and ¶), *p* < 0.001. (d) Protein expression of nuclear factor- (NF-) *κ*B. ∗ versus other groups with different symbols (†, ‡, §, and ¶), *p* < 0.0001. (e) Protein expression of RANTES. ∗ versus other groups with different symbols (†, ‡, §, and ¶), *p* < 0.0001. (f) Protein expression of toll-like receptor- (TLR-) 2. ∗ versus other groups with different symbols (†, ‡, §, and ¶), *p* < 0.0001. (g) Protein expression of TLR-4. ∗ versus other groups with different symbols (†, ‡, §, and ¶), *p* < 0.0001. (h) Protein expression of interleukin- (IL-) 1*β*. ∗ versus other groups with different symbols (†, ‡, §, and ¶), *p* < 0.0001. All statistical analyses were performed by one-way ANOVA, followed by Bonferroni multiple comparison post hoc test (*n* = 6 for each group). Symbols (∗, †, ‡, §, and ¶) indicate significance at the 0.05 level. SC = sham control; IR = ischemia-reperfusion; ECSW = extracorporeal shock wave; ADMSC = adipose-derived mesenchymal stem cell.

**Figure 10:**
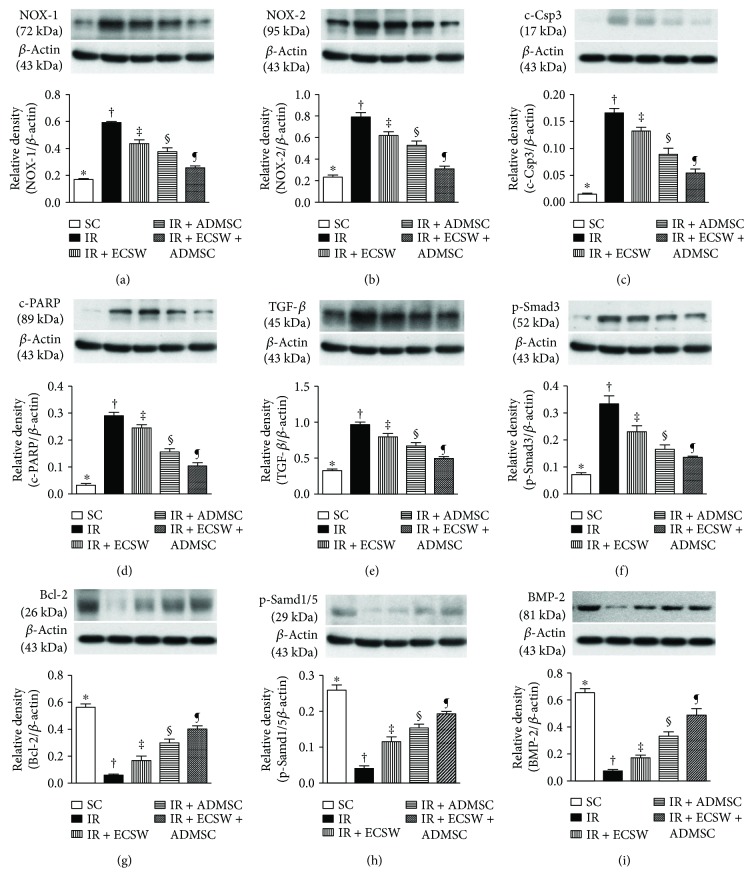
Protein expressions of oxidative-stress, apoptotic, antiapoptotic, fibrotic, and antifibrotic biomarkers in the quadriceps by day 7 after the IR procedure. (a) Protein expression of NOX-1. ∗ versus other groups with different symbols (†, ‡, §, and ¶), *p* < 0.001. (b) Protein expression of NOX-2. ∗ versus other groups with different symbols (†, ‡, §, and ¶), *p* < 0.0001. (c) Protein expression of cleaved caspase (c-Csp)3. ∗ versus other groups with different symbols (†, ‡, §, and ¶), *p* < 0.0001. (d) Protein expression of cleaved poly (ADP-ribose) polymerase (c-PARP). ∗ versus other groups with different symbols (†, ‡, §, and ¶), *p* < 0.0001. (e) Protein expression of transforming growth factor- (TGF-) *β*. ∗ versus other groups with different symbols (†, ‡, §, and ¶), *p* < 0.0001. (f) Protein expression of phosphorylated- (p-) Smad3. ∗ versus other groups with different symbols (†, ‡, §, and ¶), *p* < 0.0001. (g) Protein expression of Bcl-2. ∗ versus other groups with different symbols (†, ‡, §, and ¶), *p* < 0.0001. (h) Protein expression of p-Smad1/5. ∗ versus other groups with different symbols (†, ‡, §, and ¶), *p* < 0.0001. (i) Protein expression of bone morphogenetic protein- (BMP-) 2. ∗ versus other groups with different symbols (†, ‡, §, and ¶), *p* < 0.0001.
